# Supplementary material for: Synergy of Mutation-Induced Effects in Human Vitamin K Epoxide Reductase: Perspectives and Challenges for Allo-Network Modulator Design
Source: Int J Mol Sci. 2024 Feb 7;25(4):2043. doi: 10.3390/ijms25042043 (PMC10889538; doi:10.3390/ijms25042043)
Supplement: Supplementary file 1 [file ijms-25-02043-s001.zip › ijms-2857065-supplementary.pdf]

International Journal of
Molecular Sciences

Synergy of Mutation-Induced Effects in Human Vitamin K Epoxide Reductase: Perspectives and Challenges for Allo-Network Modulator Design

Marina Botnari and Luba Tchertanov *

Supplementary Materials

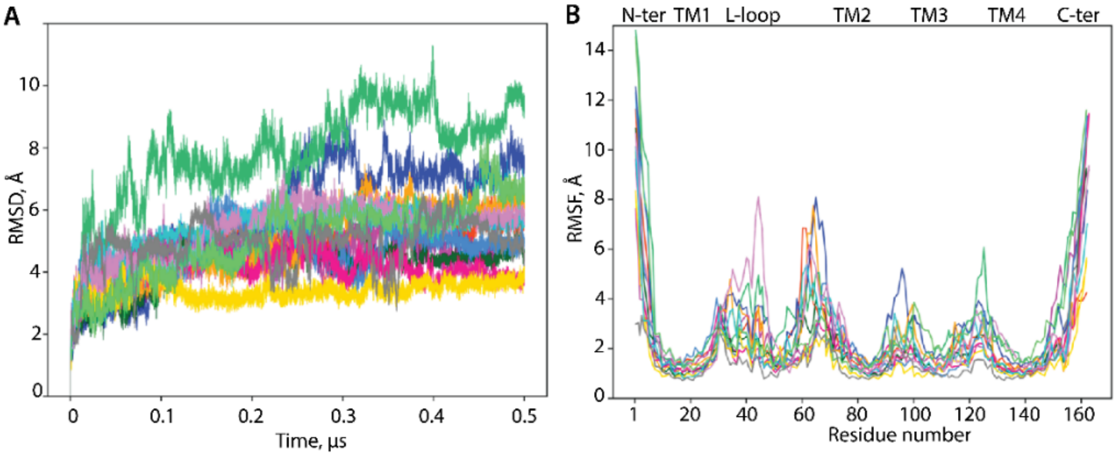

Figure S1. Characterisation of the MD simulations for the four hVKORC1 mutants compared to the native enzyme. (**A**) RMSDs from the initial coordinates computed for all Cα-atoms (right) in each protein after fitting to initial conformation. (**B**) RMSFs computed for the Cα-atoms for the MD conformation of each protein after fitting to the initial conformation (reference structure at t=0). Mutated proteins are distinguished by colour (1/2/3 replicas): VKOR^{A41S}(R) (orange red/orange/gold), VKOR^{H68Y}(R) (dark blue/blue/turquoise), VKOR^{S52W} (D) (fuchsia/pink/violet) and VKOR^{W59R} (D) (dark green/sea green/lime); VKOR^{WT}(grey).

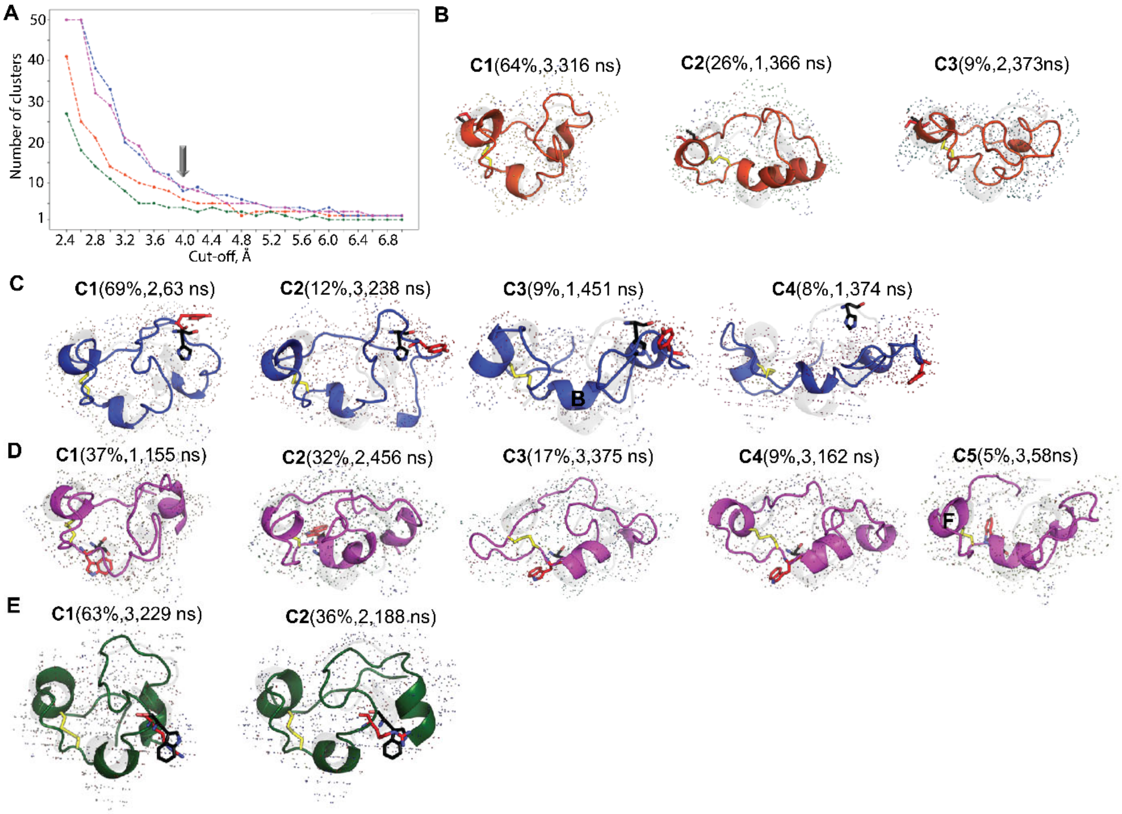

Figure S2. Ensemble-based clustering of MD conformations of the L-loop from hVKORC1 mutants. (**A**) Number of clusters obtained for each mutant using concatenated trajectories. Clustering was performed on each 10-ps frame of every trajectory using cut-off values that varied from 2.4 to 7.0 Å, with a step of 0.4 Å. The cut-off value of 4.0 Å was estimated as the optimal. (**B-E**) Representative conformations of the L-loop from clusters (C) with population ≥5%. The population of each cluster is given in brackets (in %), together with the replica number (in bold) and the time (in ns) over which the representative conformation was recorded. Mutated proteins are distinguished by colour: hVKORC1^{A41S} (orange red), hVKORC1^{H68Y} (dark blue), hVKORC1^{S52W} (fuchsia), and hVKORC1^{W59R} (dark green); hVKORC1^{WT}(gray). The L-loop of mutants is shown as coloured ribbons superposed with the L-loop of hVKORC1^{WT} presented in grey cartoon with a meshed surface. Disulphide bridges C43–C51 are drawn as yellow sticks, and the mutated and native residues are shown as red and black sticks respectively.

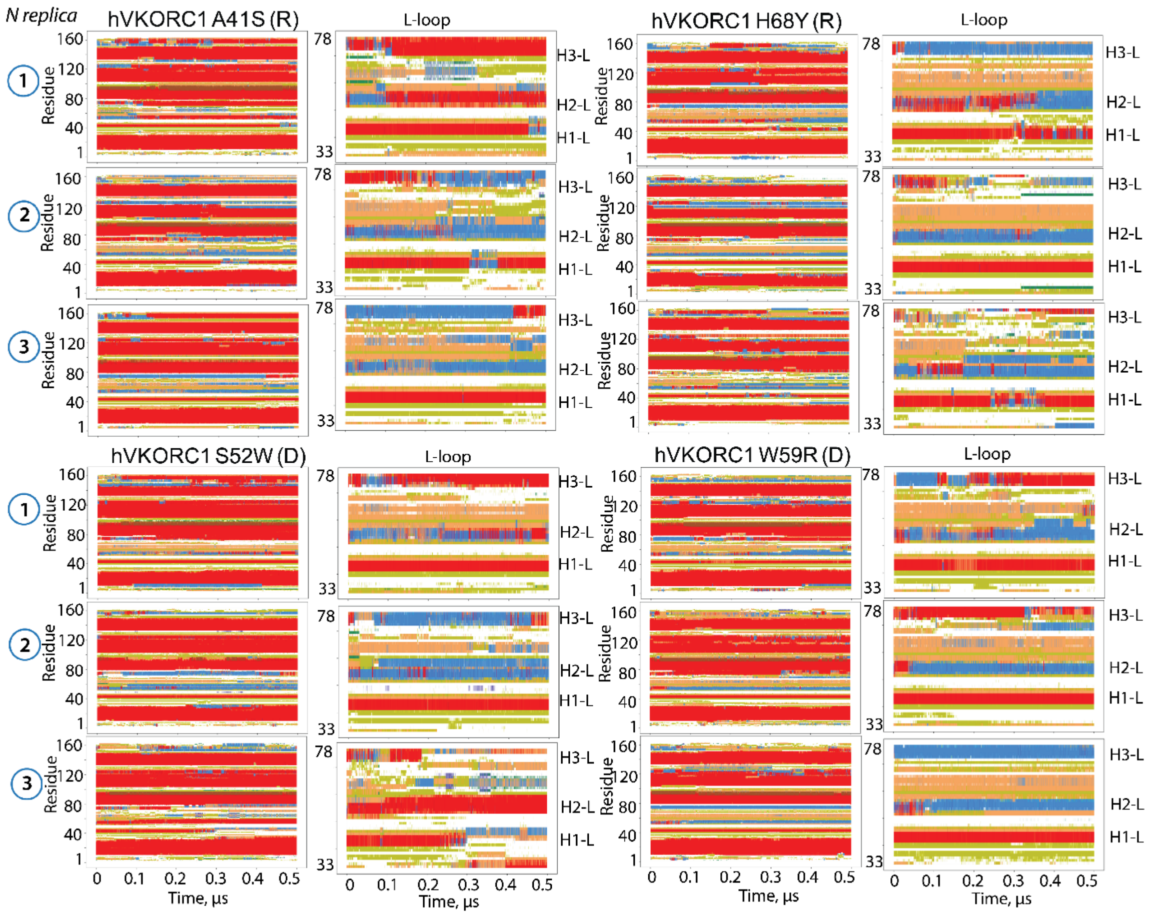

Figure S3. Folding of L-loop from hVKORC1 mutants in the inactive state. The time-dependent evolution of the secondary structure of each residue, as assigned by the Define Secondary Structure of Proteins (DSSP) method: α-helix is in red, 3^{10}-helix is in blue, turn is in orange, and bend is in dark yellow is shown for the full-length proteins (1st and 3rd columns) and L-loop (2nd and 4th columns). L-loop helixes (H1-L, H2-L, H3-L) are denoted. The index of replica (1-3) is encircled.

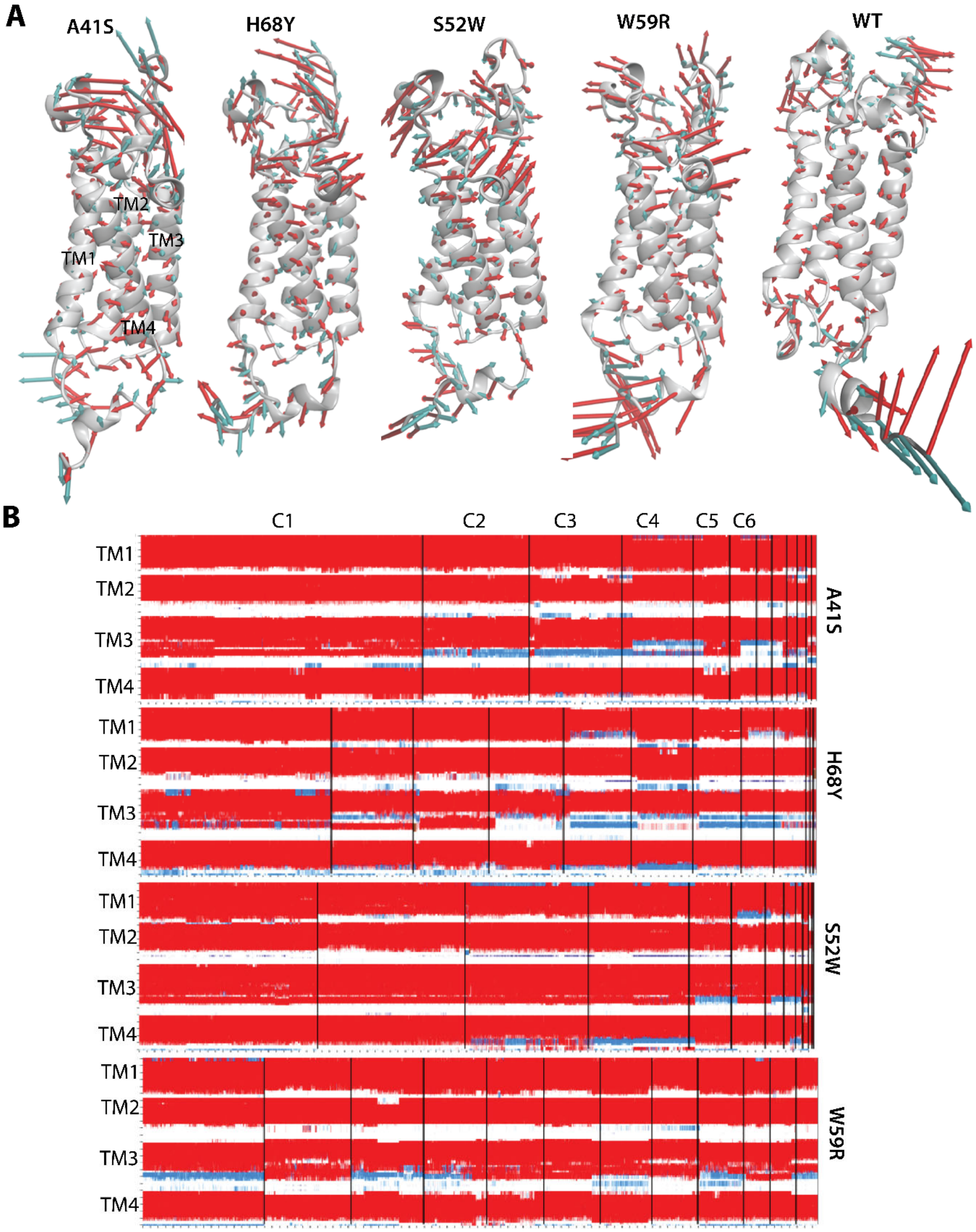

Figure S4. Principal Component Analysis (PCA) of hVKORC1. (**A**) PCA modes calculated for the concatenated MD trajectory of each hVKORC1 full-length mutant (1-163 residues) after least-square fitting of the MD conformations to the average conformation. Atomic components in the first PCA modes are drawn as red (1st mode) and blue (2nd mode) arrows projected onto the respective average structure shown as cartoon. Only motion with an amplitude ≥4 Å is considered. (**B**) Clustering of the TMD conformations using their secondary structure content. The α- and 3^{10}-helices are in red and blue respectively.

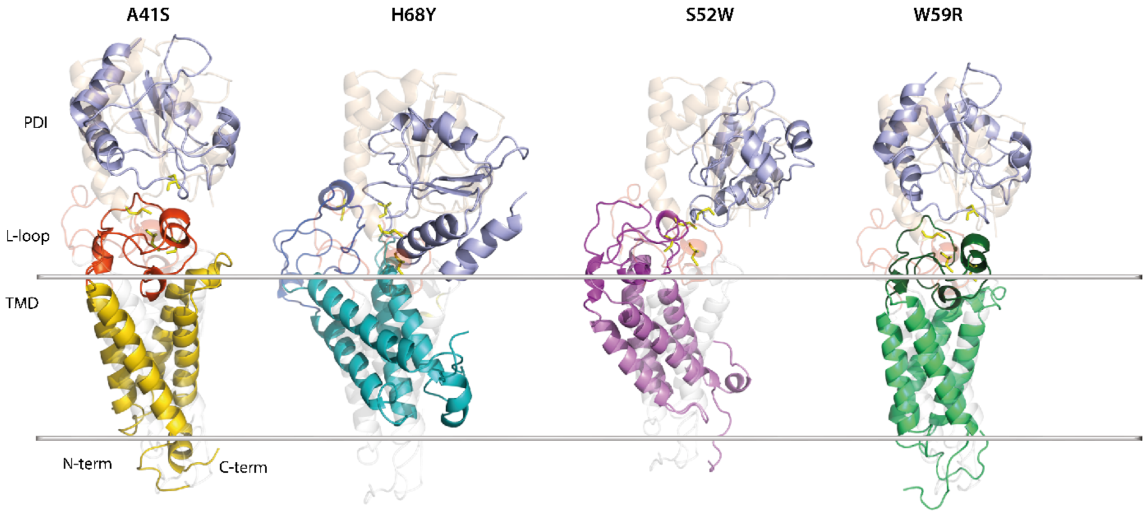

Figure S5. Computational protein–protein docking of PDI (ligand) onto hVKORC1 (target) performed with HADOCK using an information-driven method. The best solution (most populated cluster C1) is shown for each hVKORC1 mutant and superimposed into the model of PDI/ hVKORC1^{WT}. Proteins are presented as a carton with PDI in lilac and hVKORC1 mutants are distinguished by colour. In PDI/ hVKORC1^{A41S} and PDI/ hVKORC1^{W59R} models, the TMD and L-loop of hVKORC1 are denoted in yellow and red, and green and dark green, respectively. Disulphate bonds are in sticks. A possible boundary of the membrane is shown by grey lines.

Table S1. Docking of PDI on hVKORC1 mutants with HADDOK.

**hVKORC1^{A41S}/PDI**									
**Metric**	**C1**	**C2**	**C3**	**C4**	**C5**	**C6**	**C7**	**C8**	**C9**
**Score^{i}**	**-72.7±7.2**	**-57.7±3.2**	**-52.8±1.6**	**-75.4±4.7**	**-73.9± 5.3**	**-63.5±4.3**	**-47.5±7.3**	-64.2±3.6	-20.7±4.2
Size^{ii}	35	28	21	16	13	11	8	6	5
RMSD^{iii}	2.3±0.8	13.2±0.0	12.5±0.3	13.8±0.4	15.6±0.7	9.8±0.7	10.8±0.8	2.9±0.6	11.8±0.7
VdW^{iv}	-22.4±1.5	-28.3±2.6	-23.9±2.7	-22.8±5.0	-17.9±3.2	-16.5±5.9	-6.0±2.9	-13.9±3.1	-8.3±3.5
EE^{v}	-195.6±31.6	-81.2±28.4	-49.8±20.3	-169.9±20.8	-155.6±41.6	-201.3±22.9	-192.9±33.0	-246.2±21.8	-31.7±35.5
DE^{vi}	-11.3±2.6	-15.9±1.3	-19.1±1.2	-18.7±2.5	-25.2±3.6	-8.8±3.5	-3.9±2.9	-2.3±3.7	-9.2±2.3
RVE^{vii}	0.0±0.0	27.2±2.5	0.9±0.5	1.3±0.8	3.4±2.0	20.0±11.6	10.5±11.4	12.2±12.2	32.5±3.1
BSA^{viii}	789.6±103.1	865.2±40.5	734.7±77.7	913.5±52.4	681.1±101.8	814.2±48.1	627.2±71.3	712.5±129.6	268.8±19.2
Z-Score	-1.0	-0.0	0.3	-1.1	-1.0	-0.4	0.6	-0.4	2.2
S-S	14.9	13.7	11.0	11.6	9.8	10.4	11.5	16.9	13.4

Notes: ^{i}HADDOCK score; ^{ii}Cluster size; ^{iii}RMSD from the overall lowest-energy structure; ^{iv}Van der Waals energy; ^{v}Electrostatic energy; ^{vi}Desolvation energy; ^{vii}Restraints violation energy; ^{viii}Buried Surface Area; ^{IX}S-S distance (Å).
